# Efficacy of oral Vitamin D supplementation in reducing body mass index and lipid profile in adolescents and young adults in Colombia: A pilot clinical trial protocol nested in the SIMBA cohort

**DOI:** 10.1097/MD.0000000000021722

**Published:** 2020-08-28

**Authors:** Norma C. Serrano, Sandra L. Romero, Diana P. Suárez, Lyda Z. Rojas, Edna Magaly Gamboa-Delgado, Mario Forero, Elizabeth Guio, Doris Cristina Quintero-Lesmes

**Affiliations:** a Fundación Cardiovascular de Colombia, Floridablanca; b Universidad Industrial de Santander, Bucaramanga; c Hospital Internacional de Colombia, Piedecuesta, Colombia.

**Keywords:** body composition, calcidiol, cholesterol, obesity, triglycerides, Vitamin D, weight loss

## Abstract

**Background:**

In recent years, the role of vitamin D (VD) as a protective factor in cardiovascular disease has been recognized. Thus, there is a need to study the effect of vitamin D supplementation in the control of different cardiovascular risk factors and metabolic syndrome, especially in young populations where few studies have been conducted.

**Methods:**

Pilot study of a randomized, parallel two-arm, triple-blind clinical controlled trial in 150 adolescents and young adults in the city of Bucaramanga-Colombia. The intervention group will receive 1000 IU of VD and the control group 200 IU of VD daily for 15 weeks. The main outcomes are: serum calcifediol levels (25(OH) D), body mass index and lipid profile; secondary outcomes are complementary to the previous ones (skin folds, waist-hip ratio). Other variables will be analyzed such as assessment of dietary intake, physical activity, sun exposure, cigarette and tobacco consumption and compliance with VD supplementation.

**Discussion:**

This study is innovative since there is little evidence from clinical trials in adolescents and young adults; similar studies are not known in our context. The results of this study may facilitate the recommendation of oral vitamin D supplementation in the population of interest. In addition, it is a low-cost and easy-to-apply intervention that could contribute to the formulation and implementation of health policies.

**Trial registration:**

NCT04377386

## 1 Introduction

The main and classic role of Vitamin D (VD) is the mineralization of the bone, regulating the activity of osteoblasts and facilitating the absorption of calcium and phosphorus from the intestines.^[1]^ Low VD levels have been associated to an increase in the incidence and mortality caused by chronic diseases such as cardiovascular diseases (CVD) among others, and their risk factors such as diabetes, obesity and dyslipidemia.^[2–4]^

These findings are clinically important because vitamin D deficiency (VDD) is favored by a sedentary lifestyle and excessive consumption of high-calorie foods, which in turn leads to obesity.^[5,6]^ Since this is a multifactorial condition, described as a phenotype of numerous pathologies, is one of the most serious public health concerns in the 21st Century and is not alien to the child and adolescent population.^[7]^

Specifically, obesity increases constantly in the child population becoming a threat by the risk of developing a number of chronic diseases in adult life, particularly CVD.^[8]^ It is estimated that more than 42 million children worldwide had overweight or obesity in 2016.^[9]^ Colombia is not alien to this problematic; by 2015, 24.4% of school children and 17.9% of adolescents had excess weight.^[10]^

Given this scenario, in 2006 a group of researchers in Bucaramanga, Colombia, set out to study the risk factors for metabolic syndrome (MS) in a group of children between 6 and 10 years of age and to initiate the SIMBA project (longitudinal study for the evaluation of cardio metabolic risk in young people in Bucaramanga).^[11,12]^ A prevalence of MS of 9.5% was determined on the baseline (95% CI: 8.0–11.3%)^[11]^ following the criteria of the Adult Treatment Panel III (ATP III) and 8.0% (95% CI: 6.6–9.7%) based on the International Diabetes Federation (IDF). Subsequently, the SIMBA II project was conducted between 2013 and 2017, which established a prevalence of MS of 13.1% and 14.8% according to ATP III and IDF, respectively; evidencing an increase in the prevalence of MS between 3.7% and 6.8% from school age to adolescence, in accordance with each criterion.^[13]^

Consequently, research has recently begun on the efficacy of VD supplementation in children. Thus, Rajakumar et al, found that the correction of VD levels in overweight and obese children, by administering vitamin D3 with 1000 or 2000 IU/day versus 600 IU/day, had no statistically significant effect on measures of arterial endothelial function or stiffness, systemic inflammation or lipid profile, however, they were able to evidence a reduction in blood pressure, glucose levels and improvement in insulin sensitivity, suggesting that optimization of the VD status of children can improve their cardiovascular health.^[14]^

Likewise, Marwaha et al, showed that VD supplementation with the three daily doses of vitamin D3 (600, 1000, and 2000 IU) lead to a significant increase on the serum levels of 25 (OH) D in school children with VDD and the secondary hyperparathyroidism decreased from 31.7% to 8.4% after supplementation.^[15]^

A significant aspect to consider in the vitamin D supplementation interventions is that they are low cost, easy to implement and very safe; consequently, if a positive effect is achieved, a problem of great impact and increasing prevalence, such as the metabolic syndrome and cardiovascular risk, would be intervened.

## 2 Objective

To evaluate the efficacy of oral vitamin D supplementation in the reduction of the body mass index (BMI) and the lipid profile in adolescents and young adults of the SIMBA cohort in Bucaramanga, Colombia.

## 3 Methods

Design: Two-arm, triple-blind, parallel randomized controlled clinical trial.

### 3.1 Population

**Figure 1:**
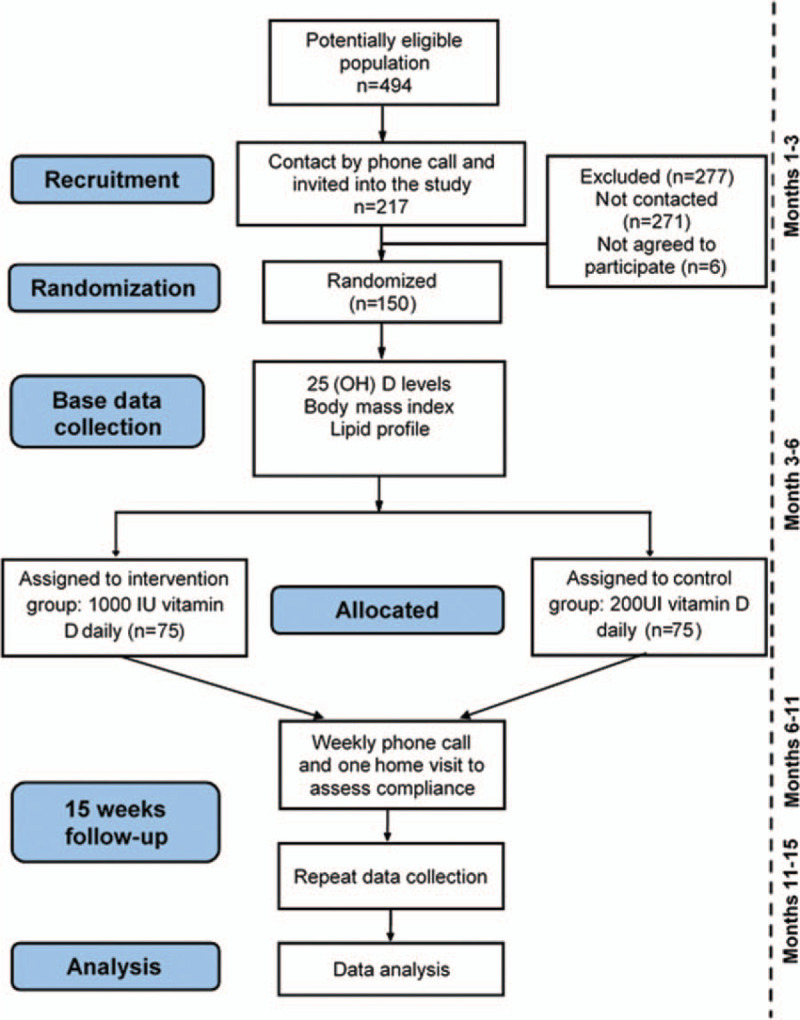
Population eligible.

The participants in the study are adolescents and young adults from the SIMBA cohort, initiated in year 2006 with a total of 1282 children between the ages of 6 and 10 years in the city of Bucaramanga, Colombia.^[11]^ Between 2013 and 2017 the SIMBA II project was carried out, which managed to contact and follow up 494 adolescents.^[13]^ Finally, in 2019, 217 participants were re-contacted by telephone. The latter constitute the population eligible for this study protocol (Fig. 1).

### 3.2 Eligibility criteria

Inclusion criteria

- Belonging to the SIMBA cohort.
- Age ≥ 18 years.
- Re-contact consent from previous studies.

Exclusion criteria

- Medical diagnosis of diabetes mellitus, clinically manifest endocrinopathies, acute or chronic infectious liver disease, and kidney disease.
- Adolescents or young adults subject to treatment with steroids or hormones (except levothyroxine) up to 1 month before lab sample taking.
- Current supplementation with VD (any presentation).

### 3.3 Sample size

The simple size was calculated considering the following parameters: an expected difference in the study outcomes (overweight, obesity, and alteration of the lipid profile) of 20% between the intervention group and the control group; a power of 90%, an alfa of 5%, an intervention group/control group ratio of 1:1, and a 20% adjustment for losses to follow-up, resulting in a sample of 270 participants (n = 135 intervention and n = 135 control group). The OpenEpi software was used for the calculation. Since this is a pilot study and considering the feasibility, the research team decided on a sample size of 150 participants (n = 75 intervention group and n = 75 control group).

### 3.4 Randomization and blinding

The randomization process will take place in 2 stages. An engineer from the data central will conduct the randomization of the list of the 217 potentially eligible participants by means of a computer program with random numbers and it will select 150 individuals to participate in the study. Subsequently, a nurse will make phone calls to schedule appointments and formalize the participation in the study, the signature of the informed consent, and the taking of baseline measures. Once these stages are concluded, the assignment of treatment in real time will be made with the support of a computer program. The procedure will take place by means of a single assignment sequence that determines the assignment code for each participant's treatment. This process allows all participants in the study to be assigned to any of the treatment groups. The intervention group will receive 1000 IU of VD while the control group will receive 200 IU of VD.

Supplements will be packaged in individual packages with the total number of doses per participant (105 doses). The coding of the packages will be in charge of the laboratory in charge of providing the supplements, which in turn will send to the designated person at the data center by institutional e-mail, a document with the list of codes and the dose of the supplement.

The 1000 IU and 200 IU doses of VD will be provided in white containers and will be identical in terms of physical and organoleptic characteristics, to ensure that both participants and investigators are blinded to the treatment. To maximize adherence to the intervention, participants will be required to cross out daily supplementation on a calendar with the study period, which they will be required to return at the end of the study.

The results of the assignment will only be known by the systems engineer at the data center. In other words, this information “will not be visible” to the coordinating nurse, laboratory personnel in charge of processing biological samples, pediatrician, principal investigator of the study, or the epidemiologist in charge of quality control and data analysis. This will ensure the masking of the people in charge of measuring the outcomes, administering the intervention and analyzing the study data.

### 3.5 Recruitment

For recruitment, a study nurse in charge of enrollment will contact by phone the 217 successful participants of the third re-contact, explain the objective of the study and inquire about the interest to participate; if there is a positive response, inclusion and exclusion criteria will be verified. A list of eligible participants will be generated from the above process and sent to the person in charge of the randomization process.

After the randomization process, the study nurse will receive from the data center the list of the 150 participants selected, who will be invited to participate in the clinical trial. The nurse will call them on the pone for a face-to-face appointment where the details of the study will be informed, doubts will be clarified, and the informed consent form will be signed; a copy of the form will be given to the participant.

During that appointment, the collection of demographic and clinical data will take place, as well as a blood simple for further measurement of VD, lipid profile and glycaemia; the anthropometric nutritional, dietary and physical activity assessment will also be carried out. Once the baseline measurements are taken, the assignment to the intervention will be made in real time through software designed for the study.

Finally, the supplementation intervention will be explained in detail and the package corresponding to the participant's code will be delivered according to the process of assignment to the group (intervention/control), it will be clarified that the supplementation will last 15 weeks and then the second meeting will be scheduled to carry out again the measures taken at the beginning of the study. At the end, a third meeting will be scheduled for the delivery of results and recommendations.

### 3.6 Interventions

The intervention group will receive 1000 IU of VD and the control group 200 IU of VD daily for 15 weeks. All participants will be given 105 doses of VD. The supplementation will be provided by the laboratory *Farma de Colombia* and is commercially named as Farma D. Its commercial presentation is soft gelatin capsules.

The 75 participants assigned to the intervention group will take one (1) 1000 IU VD capsule daily for 15 weeks. The daily dose selected is based on the amount required for replenishment over a period of approximately 3 months, and is well below the intake levels associated with toxicity.^[16]^ On the other hand, the 75 participants in the control group will take 1 capsule of VD of 200 IU daily for 15 weeks, since the benefits of VD for the health of people have been demonstrated, this being the minimum recommended dose for children. The above, considering that it is unethical for the control group not to receive any type of supplementation.

The participants in both the intervention and the control group must record in a calendar the time of the daily intake of the supplement during the 15 weeks.

### 3.7 Follow-up

Follow-up will be done 15 weeks after the start of the study (after the end of the intervention).

In addition, all participants will be followed up with a telephone call at weeks 1, 2, 5, 7, and 12. These calls will inquire about any “potential” adverse effects related to the intervention and assess adherence to DV sampling by means of a self-reporting instrument (Table 1).

**Table 1:**
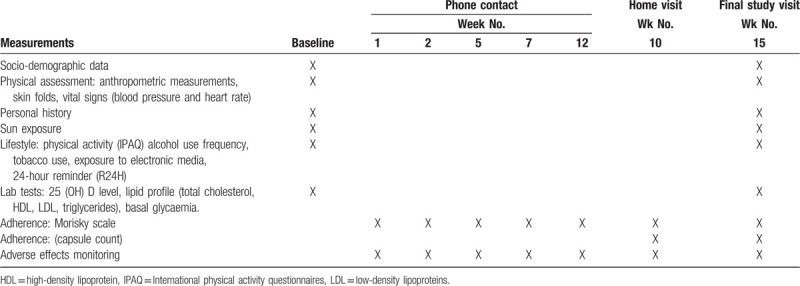
Summary of measurement and follow-up activities.

### 3.8 Safety considerations

The recommendation of vitamin D in the diet for the Colombian population (males-females) is 600 IU/day, a value equivalent to a nutritional density of 40 IU/1000 Kcal. In adolescence, plasma levels decrease and needs increase; however, the recommendation is the same as for adults.^[17]^

Normally there is no toxicity by VD since it is a fat-soluble vitamin and the organism excretes it regularly. Thus, the causes of toxicity are not related to the diet or sun exposure, but to the chronic consumption of high doses of VD supplements. This is because the body regulates the amount of vitamin D produced by sun exposure and, and even fortified foods do not have large quantities of this vitamin.

Although the recommended doses are below 2000 IU, the maximum level of daily consumption which is considered tolerable is 4000 IU for people between the ages of 1 and 18, and 10,000 IU for people over the age of 19.^[18]^ On the other hand, the doses that have been documented to lead to toxicity are above 40,000 IU to 50,000 IU per day.^[16,17]^ This is considered as potentially severe condition whose symptoms are associated to secondary hypercalcemia and are mainly of a gastrointestinal nature (anorexia, nausea, vomit, constipation, diarrhea, abdominal pain) and polydipsia, polyuria, muscular weakness and fatigue, among the most relevant.^[19]^

Therefore, the supplementation with an orally administered dose of 1000 IU/day of vitamin D is completely safe for the participants and it does not have adverse effects.

### 3.9 Outcomes

*Primary outcome:* These are biochemical and anthropometric measurements that allow to respond to the purpose of the study. These include:

- Total BMI. This requires taking the size in meters (m) and weight in kilograms (kg).
- Lipid profile: Measurements will be made of the serum levels of total cholesterol, low-density lipoprotein (LDL), high-density lipoprotein (HDL), and triglycerides.
- Serum levels of 25(OH) D.

*Secondary outcomes:* These are complementary measurements for the primary outcomes.

- Waist-hip ratio: determines the intra-abdominal fat levels and is obtained by dividing the waist perimeter by the hip perimeter.
- Skin folds: These are taken for the indirect estimation of body fat percentage by means of measuring the bicipital, tricipital, abdominal, and subscapular folds.
- Fasting glycemia.

### 3.10 Data collection and statistical analysis

Taking samples, measurements, and data collection will be done by following the operating procedures manual which describes in detail each one of the procedures. Data will be digitally uploaded in real time by means of a digital questionnaire.

#### 3.10.1 Body mass index

The procedure will be conducted by a trained nurse. Participants must be fasting, wearing minimum clothes, without shoes, belts or other accessories that may alter the measurements. The measure of body weight will be in kilograms (kg) to one decimal place, and the size in meters (*m*) to 2 decimal places. From these measurements the BMI will be obtained using the formula: BMI = Weight (kg) / size (m)^2^.

Size will be measured with a SECA 213 stable stadiometer, that has a measuring range of 20 to 205 cm (8–81”) with features that allow an accurate reading. Weight will be measured using a SECA 813 digital scale which has a steel frame as reinforcement, fit for people weighing up to 200 kg (440 lb); the measurement is performed twice to ensure a more reliable measurement (if there is a difference of less than 0.5 kg, the second value is recorded; if it is greater than 0.5 kg, a third one should be taken and the value closest to the latter should be recorded).

#### 3.10.2 Lipid profile

After a 10 to 12 hour fast, a laboratory assistant will draw a sample of peripheral venous blood through a puncture of the basilic cephalic vein or its medians and extract approximately 15 cc to 20 cc of blood. The processing and analysis of the samples will be done respectively through enzymatic technique, selective liquid detergent, selective detergent accelerator and Glycerol Phosphate Oxidase in the clinical laboratory of *Hospital Internacional de Colombia*, which is certified and has a high-quality registry.

#### 3.10.3 25(OH) D levels

25-hydroxyvitamin D are measured from the same blood samples drawn for lipid profile and quantified in serum by chemiluminescent microparticle immunoassay (CMIA).

#### 3.10.4 Waist–hip ratio

Measurements will be made by the trained nurse with an inelastic tape measure in centimeters (cm) to one decimal place, directly on the skin of the abdomen and bare hip or with soft garments that do not make volume or put pressure on the tissues. The person should have both of his or her hemispheres aligned and preferably be barefoot or wear flat shoes.

The circumference of the waist shall be measured at the midpoint between the edge of the lower sack (10th rib) and the upper edge of the iliac crest, without adjusting or stopping touching the skin. With the person relaxed, 2 measurements shall be taken without removing the tape, just checking the position. If the difference between the two measurements is less than or equal to 1 cm, the average of the 2 will be taken and if the difference is greater, a third measurement will be taken and the intermediate value between the three measurements will be recorded.

For the measurement of the hip, the knees and the heels must be together; the most prominent part of the hip on both sides shall be located and the tape measure shall be passed following the same recommendations as for the circumference of the waist.

#### 3.10.5 Skinfolds

These measurements will be taken by a trained nurse using a Harpender skinfold caliper. The skinfold will be taken with the index and thumb, and the caliper will be placed 1 cm inwards the fold. It will be applied for 3 seconds before taking the reading while the needle stops oscillating.

- *Bicipital fold:* It shall be taken parallel to the longitudinal axis of the arm above the acromial-radial midline, on the anterior face of the arm above the mid-portion of the bicep.
- *Tricipital fold:* It shall be taken parallel to the longitudinal axis of the arm on the acromial-radial midline, in the posterior region of the arm on the mid-portion of the triceps.
- *Abdominal fold:* it shall be taken vertically, parallel to the longitudinal axis of the body, at the level of the navel, approximately five centimeters from the navel on the right side of the rectus abdominis
- *Subscapular fold:* the lower angle of the scapula shall be felt to determine the most prominent lower point; the fold shall be taken two centimeters from the lower angle of the scapula, in an oblique direction, downwards and outwards, forming an angle of 45 degrees with the horizontal axis.

The percentage body fat (PBF) is taken for the indirect estimation by means of measuring the bicipital, tricipital, abdominal, and subscapular folds. Percentage of body fat (%BF) calculated with the Siri equation for four skin folds (SF), like so: % BF SIRI = ((4.95/ D) − 4.5) × 100. Body density (D) is obtained using the linear regression equation proposed by Durnin and Womersley (C and D). D = C − M × log10 ∑ 4SF (bicipital, tricipital, abdominal, and subscapular).^[20]^

#### 3.10.6 Fasting blood glucose level

After a 10 to 12 hour fast, blood glucose levels are measured in serum from the same blood samples drawn for lipid profile, using the hexokinase/G-6-PDH technique.

### 3.11 Statistical analysis

An analysis will be made by intention to treat. The description of the categorical variables will be made by means of absolute and relative values. Quantitative variables that present a normal distribution in Shapiro Wilk's test will be reported as mean and standard deviation, otherwise the median and interquartile range will be presented. The comparison of the basal characteristics of the study groups (intervention and control) will be done using the ratio comparison test (Chi-square or Fischer's exact test) and the Student's t-test or Mann-Whitney's U-test.

Quantitative outcome variables will be contrasted through paired t-tests or Wilcoxon tests for quantitative variables, methods of longitudinal data analysis will also be explored and for categorical ones the Mc Nemar test will be used. Statistical significance will be considered for all hypothesis tests when the *P* value is less than .05.

### 3.12 Ethics approval and consent to participate

The research will be carried out following the principles established in the Good Clinical Practice in Clinical Trials GPC/ICH. It is considered a minimum risk study according to resolution 008430 of October 4, 1993 of the Colombian Ministry of Health, since it includes the administration of a commonly used vitamin supplement with a wide therapeutic margin and for its administration the indications, doses and routes of administration established by the Institute for Drug and Food Surveillance (Invima, acronym in Spanish) are considered. The vitamin supplement is supplied by the laboratory Farma de Colombia, commercially known as Farma D, whose presentation is in soft gelatin capsules and with registration number 2017 M-0012231-R1.

The processing of personal and clinical data and biological samples will be handled according to the Habeas Data Act (Act 1266 of 2008) of the government of Colombia.

The study was reviewed and endorsed by the Technical Scientific Committee (CTC) of *Fundacion Cardiovascular de Colombia* (FCV) according to minute N°133 of May 2018 and has the approval of the Research Ethics Committee (IEC) of the FCV according to minute N°480 of July 16, 2019.

The study nurse will explain the objectives, importance, risks, benefits of the research and the confidentiality of the data. Written informed consent will be given to study participants and for participants under the age of 18 we will obtain from a parent or guardian.

## 4 Discussion

The role of vitamin D as a protector against cardiovascular disease is widely recognized.^[2,3]^ For consideration, vitamin D supplementation is being widely studied to determine its effect on the control of cardiovascular risk factors and metabolic syndrome, including obesity, dyslipidemia, and impaired blood glucose.^[5]^

Studies on the effect of supplementation in adolescents and young adults are scarce; however, the favorable effect of supplementation in the serum levels of 25 (OH) D in school-age children with VDD and the reduction in blood pressure, the concentration of fasting glucose, and improvements in sensitivity to insulin in children with overweight and obesity is widely known.^[14,15]^ Notwithstanding this, more clinical trials are required to confirm these findings.

This research is innovative considering that there are few studies on adolescent and young adult populations and that these have not been carried out in Latin America.^[14,15]^ Consequently, if this trial provides positive results, it will be possible to recommend oral vitamin D supplementation for the reduction of BMI and lipid profile in the studied population. In addition, it is a low-cost and easy-to-apply intervention that could contribute to the formulation and implementation of health policies.

## Author contributions

NCS, EMG, MF, EG, and DCQ conceived the study and requested the funding; LZR, SLR, DPS, and DCQ made adjustments and refined the protocol; SLR, LZR, and DPS drafted the paper; NCS, EMG, EG, and MF reviewed the manuscript. All authors read and approved the manuscript's final draft.
